# The Defense Responses of *Eucalyptus* to Insect-Feeding and Leaf-Clipping Stresses

**DOI:** 10.3390/life16040604

**Published:** 2026-04-05

**Authors:** Cong Chen, Zihui Zhang, Wenxin Lin, Meiling Xu, Zhaoyan Lin, Keling Zheng, Tuerxun Ailiminuer, Qijing Wu, Xingyu Chen, Hua Chen

**Affiliations:** School of Life Sciences, Zhaoqing University, Zhaoqing 526061, China; zihuizhang8@163.com (Z.Z.); 13592803193@163.com (W.L.); meilingxu2024@126.com (M.X.); linzhaoyan123@126.com (Z.L.); 13502993483@163.com (K.Z.); amy070923@163.com (T.A.); qijingwu79@163.com (Q.W.); 16675827107@163.com (X.C.)

**Keywords:** leaf-clipping stress, *Buzura suppressaria*, pest-feeding stress, defense enzymes, defense compounds, *Eucalyptus* plantation

## Abstract

*Eucalyptus* spp. are the most important timber and pulpwood species in southern China. This tree species is frequently and severely damaged by the leaf-eating pest *Buzura suppressaria*, which significantly impairs photosynthesis and hinders the healthy and sustainable development of the *Eucalyptus* industry. To investigate the defensive responses of *Eucalyptus urophylla* × *Eucalyptus grandis* to pest (*B. suppressaria*)-feeding and leaf-clipping stress, this study measured the temporal changes in defense enzyme activities and defense compounds in *Eucalyptus* under conditions of pest-feeding and leaf-clipping stresses, aiming to provide a theoretical basis for resistance breeding in *Eucalyptus*. The results show that pest-feeding and leaf-clipping stress groups significantly affected the peroxidase (POD), polyphenol oxidase (PPO), and phenylalanine ammonia-lyase (PAL) activities in *Eucalyptus* leaves. Within a short period after stress (3 h), POD activity was significantly reached 444.83 U by leaf-clipping stress, whereas it was significantly inhibited (34.83 U) by pest-feeding stress. PPO activity was significantly enhanced to 95.25 U under pest-feeding stress within 3 h, while leaf clipping induced a lower level of PPO activity (58.75 U). PAL activity was significantly induced to 474.38 U by leaf-clipping stress at 3 h, whereas pest-feeding stress resulted in a moderate increase to 238.00 U. Both pest-feeding and leaf-clipping stresses had significant effects on the contents of defense compounds in Eucalyptus leaves. Within a short period (3 h), both leaf-clipping and pest-feeding stresses significantly induced the accumulation of salicylic acid (0.226 μg/g and 0.326 μg/g, respectively), jasmonic acid (0.239 μg/g and 0.278 μg/g, respectively), and tannin (0.581 μg/g and 0.657 μg/g, respectively). The POD activity and salicylic acid content were identified as the primary factors in *Eucalyptus* responses to pest-feeding and leaf-clipping stresses. In conclusion, biotic (pest-feeding) and abiotic (leaf-clipping) stresses can induce higher activities of related defense enzymes while also promoting the synthesis of greater quantities of defensive chemical compounds, thereby enhancing the resilience to biotic and abiotic stresses in *Eucalyptus*. This study provides important practical guidance for insect-resistant *Eucalyptus* breeding and implementing integrated pest management strategies.

## 1. Introduction

Plant stress resistance is a key factor enabling plants to thrive. Induced resistance in plants, also known as indirect defense responses, includes induced insect or stress resistance. This refers to the insect-resistant characteristics induced in plants in response to insect feeding attacks or stress resistance developed in response to abiotic stress, the purpose of which is self-protection [1]. Induced stress resistance in plants encompasses changes in multiple aspects, such as morphological characteristics; physiology; and biochemistry, including alterations in the activity of defense enzyme systems and changes in the content of defense substances, such as salicylic acid, jasmonic acid, condensed tannins, and flavonoids [2]. Induced resistance in plants can be categorized into local and systemic induced resistance characterized by broad-spectrum activity, dynamism, and non-specificity. The induced defense process can be summarized into three stages: systemic monitoring, signal recognition, and defense substance production [3]. Chemical substances constitute the most important factor in the defense mechanisms induced by biotic or abiotic stress in plants, such as the synthesis and release of resistance-related proteases and defense substances. These primarily activate the plant’s own immune system through induced resistance-related substances, ultimately achieving defense goals [2]. When plants are subjected to stress, the enzyme systems that control material metabolism in the plant body respond most rapidly, particularly the key enzyme systems involved in the synthesis of secondary metabolites for defense substances. Examples include polyphenol oxidases (PPOs), phenylalanine ammonia-lyase (PAL), and peroxidases (PODs). These three key enzymes are also referred to as defense enzyme systems, and they play a significant role in plant defense against insects [4]. Plant tannins are phenolic secondary metabolites that can specifically bind to proteins, thereby inhibiting the proteolytic enzyme activity. They can also form complexes with starch, affecting the insect’s ability to break down or digest nutrients such as starch and proteins [5]. Many studies have shown that flavonoids have strong toxic effects on insects [6,7]. After recognizing insect feeding, plants can also activate multiple signaling pathways within their bodies. Jasmonic acid (JA), salicylic acid (SA), and tannins play important roles in plant defense against environmental stresses. For example, studies have shown that exogenous application of salicylic acid can enhance plant tolerance to drought stress by increasing the activities of antioxidant enzymes such as superoxide dismutase (SOD), ascorbate peroxidase (APX), and catalase (CAT) [8]; jasmonic acid signaling pathways have been demonstrated to mediate herbivore resistance in tea plants by regulating the expression of JA biosynthesis genes such as CsLOX2, CsLOX3, and CsAOC3/4 [9]; and tannins, as phenolic secondary metabolites, accumulate in response to insect feeding and can inhibit insect digestive enzymes by binding with proteins [10] such as those involving SA, ethylene (ET), and hydrogen peroxide (H_2_O_2_). This leads to the accumulation of plant defense compounds, the expression of defense genes, and the release of volatile substances, further enhancing plant resistance to herbivorous insects [11].

*Eucalyptus* refers collectively to species within the genera *Eucalyptus*, *Angophora*, and *Corymbia* of the *Myrtaceae* family. Native to countries such as Australia, Papua New Guinea, Indonesia, and the Philippines, there are a total of 945 species and varieties [12]. *Eucalyptus* is characterized by rapid growth, high yield, and good adaptability, and it is recommended by the Food and Agriculture Organization of the United Nations as one of the world’s three major fast-growing and high-yield afforestation species (*Eucalyptus*, pine, and poplar) [13]. *Eucalyptus* is the most important timber and pulpwood species in southern China. However, with the large-scale promotion and intensive management of monocultures, *Eucalyptus* leaves are frequently damaged by pests and diseases or human mechanical injury; in particular, leaves are often completely consumed by the leaf-eating pest *Buzura suppressaria*, which impedes photosynthesis and severely affects the healthy and sustainable development of the *Eucalyptus* industry [14]. *Buzura suppressaria*, belonging to the order Lepidoptera and family Geometridae, is also known as the *Eucalyptus* measuring worm, commonly referred to as the arched-back worm or measuring worm. It is a holometabolous insect, completing one generation through four stages: adult, egg, larva, and pupa [15,16].

Defensive toxic chemical substances produced by *Eucalyptus* in response to insect-feeding and leaf-clipping stresses mainly include phloroglucinol compounds, flavonoids, terpenoids, and tannins [17]. Compounds such as chlorogenic acid, quercetin-3-O-glucuronide, hyperoside, guaijaverin, and quercetin-3-O-α-arabinosyl-2-gallate have been isolated from the leaves of *Eucalyptus urophylla* × *E. grandis* [18]. Tannins are one of the abundant chemical components in Myrtaceae plants, with hydrolysable tannins being the main structural type, along with small amounts of condensed and mixed tannins [19]. Terpenoids isolated from *Eucalyptus* mainly include triterpenes, triterpenic acids, and their ester derivatives [20]. Three solvent extracts (petroleum ether, chloroform, and ethanol) from *Eucalyptus* leaves all showed significant oviposition inhibition effects on *Phthorimaea operculella* within 24 h [21].

According to statistics from 65 countries with planted areas exceeding 5000 hm^2^, the global area of *Eucalyptus* plantations has surpassed 22.57 million hm^2^ [14,22]. Currently, there are no relevant research reports on the defense responses of *Eucalyptus* to biotic or abiotic stress. Therefore, the defense responses of *Eucalyptus* to pest-feeding and leaf-clipping stresses are analyzed in this study, with the aims of enriching our theoretical understanding of plant defense strategies against insect feeding and providing an important scientific basis for insect-resistant *Eucalyptus* breeding and integrated pest management.

## 2. Materials and Methods

### 2.1. Test Insects

**Figure 1:**
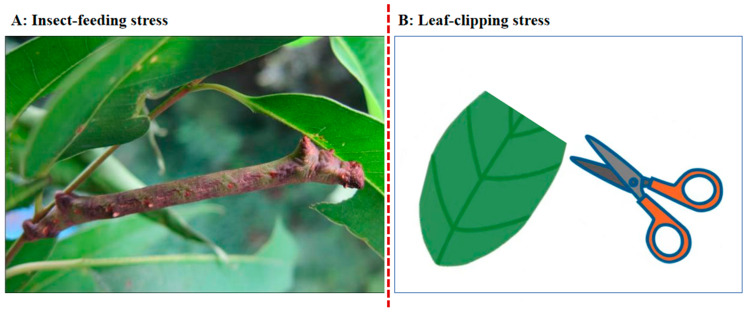
Eucalyptus responds to both biotic and abiotic stresses. (**A**) refers to insect-feeding stress, (**B**) refers to leaf-clipping stress.

*Buzura suppressaria* was collected from a plantation of *Eucalyptus urophylla* × *Eucalyptus grandis* (*Eucalyptus*) and reared in the laboratory nursery (Figure 1). Larvae hatched from the same cohort were selected for experimental trials.

Eggs laid by a synchronized batch of adults were collected. Upon hatching, the larvae were transferred individually onto potted *Eucalyptus* seedlings for rearing. When the larvae reached the third instar, the following treatment groups were established: Pest-feeding group: 3 replicates, with 20 larvae placed in each replicate to feed naturally on *Eucalyptus* leaves. Leaf-clipping group: 3 replicates, with no larvae placed; artificial leaf clipping was used to simulate feeding, with the amount of leaves clipped per hour over 24 h approximating the larval consumption rate. Control group: 3 replicates, the *Eucalyptus* leaves were healthy and had not been subjected to pest feeding or mechanical clipping.

The feeding or clipping treatments were conducted for 24 h. Afterwards, all larvae were removed from the treatment and control groups. This point was designated as the experimental starting point (0 h). Leaf tissue samples were then collected from each treatment and control group at 3, 6, and 9 h. The samples were immediately flash-frozen in liquid nitrogen and subsequently transferred to a −80 °C ultra-low temperature freezer for storage. Subsequent analyses measured the temporal changes in defense enzyme activities in *Eucalyptus*, as well as the temporal changes in the content of defense compounds induced by biotic and abiotic stress responses.

### 2.2. Temporal Changes in Eucalyptus Defense Enzyme Activities Induced by Insect-Feeding and Leaf-Clipping Stresses

#### 2.2.1. Protein Content Determination

The protein content was determined using the Coomassie Brilliant Blue G-250 staining method as described by Bradford (1976) with modifications [23]. An appropriate amount of crude enzyme solution was diluted 10-fold. An aliquot of 50 μL of the diluted enzyme solution was mixed with 750 μL of Coomassie Brilliant Blue solution, thoroughly vortexed, and allowed to stand at room temperature for 5 min. The absorbance was measured at 595 nm using a microplate reader. A standard curve was constructed using bovine serum albumin (BSA), and the protein content in the enzyme solution was calculated accordingly. All reagents were purchased from Aladdin Reagent Co., Ltd., Shanghai, China.

#### 2.2.2. Peroxidase Activity Assay

POD activity was determined using the method, which was adapted from the method described by Köksal (2011) with modifications [24].

(a) Crude enzyme extract preparation: Place 0.3 g of sample in a pre-cooled mortar. Add 1.5 mL of 20 mmol/L KH_2_PO_4_ buffer (pH 7.0), 0.03 g of insoluble polyvinylpyrrolidone (PVP), and an appropriate amount of quartz sand. Grind into a homogenate. Centrifuge at 12,000 r/min for 20 min at 4 °C. The supernatant is the crude enzyme extract.

(b) Enzyme activity assay: The reaction system consists of 4.9 mL of reaction mixture (add 28 μL of guaiacol to 50 mL of a pH 6.0, 0.1 mol/L phosphate buffer and dissolve by heating on a magnetic stirrer; after the solution cools, add 19 μL of 30% H_2_O_2_ and mix well) and 0.1 mL of the enzyme extract. Record the absorbance at 470 nm every 1 min and calculate the change in OD value over 5 min. The enzyme activity is defined as the amount that causes an increase of 0.01 in the OD_470_. All reagents were purchased from Aladdin Reagent Co., Ltd., Shanghai, China.

#### 2.2.3. Polyphenol Oxidase Activity Assay

PPO activity was determined using the method, which was adapted from the method described by Gonzalez et al. (1999) with modifications [25].

(a) Crude enzyme extract preparation: Place 0.3 g of sample in a pre-cooled mortar. Add 1.5 mL of pH 7.0, 0.05 mol/L phosphate buffer, 0.03 g of insoluble polyvinylpyrrolidone (PVP), and an appropriate amount of quartz sand. Grind into a homogenate. Centrifuge at 12,000 r/min for 20 min at 4 °C. The supernatant is the crude enzyme extract.

(b) Enzyme activity assay: The reaction system consists of 1.5 mL of reaction mixture (containing 1.35 mL of pH 7.0, 0.1 mol/L catechol phosphate buffer, and 0.15 mL of crude enzyme extract). Record the absorbance at 410 nm every 30 s, using the extraction buffer instead of the enzyme extract as the blank control. Calculate the change in OD value over 5 min. The enzyme activity is defined as the amount that causes an increase of 0.001 in the OD_410_. All reagents were purchased from Aladdin Reagent Co., Ltd., Shanghai, China.

#### 2.2.4. Phenylalanine Ammonia-Lyase Activity Assay

PAL activity was determined using the method, which was adapted from the method described by Arrieta-Guevara et al. (2010) with modifications [26].

(a) Crude enzyme extract preparation: Place 0.3 g of sample in a pre-cooled mortar. Add 1.5 mL of boric acid buffer (0.05 mol/L, pH 8.8) containing 5 mmol/L β-mercaptoethanol, 0.03 g of insoluble polyvinylpolypyrrolidone (PVPP), and an appropriate amount of quartz sand. Grind into a homogenate. Centrifuge at 12,000 r/min for 20 min at 4 °C. The supernatant is the crude enzyme extract.

(b) Enzyme activity assay: The reaction system consists of 2 mL of reaction mixture (containing 1 mL of 0.05 mol/L, pH 8.8 boric acid buffer, 0.9 mL of 0.02 mol/L phenylalanine, and 0.1 mL of enzyme extract). Incubate in a water bath at 37 °C for 1 h, then terminate the reaction by adding 6 mol/L HCl. Record the absorbance at 290 nm every 30 s, using the extraction buffer instead of the enzyme extract as the blank control. Calculate the change in OD value over 5 min. The enzyme activity is defined as the amount that causes an increase of 0.01 in the OD_290_. All reagents were purchased from Aladdin Reagent Co., Ltd., Shanghai, China.

### 2.3. Temporal Changes in Non-Volatile Defense Compound Content in Eucalyptus Induced by Insect-Feeding and Leaf-Clipping Stresses

#### 2.3.1. Salicylic Acid Content Determination

SA content was determined using the method, which was adapted from the method described by Li et al. (2021) with modifications [27]. All reagents were purchased from Yuanye Reagent Co., Ltd., Shanghai, China.

Grind the leaf tissue into a homogenate and transfer it to three 50 mL clean centrifuge tubes. Vortex for 1 min, then centrifuge at 10,000 r/min for 30 min. Collect the supernatant. Add 20 mL of pure methanol to the pellet in each tube, centrifuge again under the same conditions, and collect the supernatant. Combine the supernatants from both steps. Dry the combined supernatant using a rotary evaporator (water bath temperature 40 °C). Dissolve the residue in 15 mL of 5% trichloroacetic acid. Then, add 40 mL of a 1:1 (*v*/*v*) mixture of ethyl acetate and cyclohexane for extraction. Repeat the extraction once more. Combine the organic phases and dry again using the rotary evaporator (40 °C water bath). Redissolve the dried sample in 10 mL of solvent. Filter through a 0.45 μm organic microporous filter membrane and store at 4 °C for later use. Various solvents, including 1% acetic acid, 1% phosphoric acid, anhydrous ethanol, and methanol, were used for High-Performance Liquid Chromatography (HPLC) determination.

Precisely weigh 20 mg of salicylic acid standard and dissolve it in the chosen solvent. Transfer to a 50 mL volumetric flask, dilute to volume with the solvent, and mix well. Filter through a 0.45 μm organic microporous filter membrane to prepare a 0.4 mg/mL standard stock solution. Pipette 1, 2, 4, 8, 16, and 32 mL aliquots of the standard stock solution into separate 50 mL volumetric flasks, dilute to volume with the solvent, and mix well. A linear regression equation was established using the peak area as the *x*-coordinate and the salicylic acid concentration as the *y*-coordinate.

#### 2.3.2. Jasmonic Acid Content Determination

JA content was determined using the method, which was adapted from the method described by Zhang et al. (2008) with modifications [28]. All reagents were purchased from Yuanye Reagent Co., Ltd., Shanghai, China.

Rapidly grind *Eucalyptus* leaves into a homogenate in an ice bath using 10 mL of 80% ice-cold ethanol. Centrifuge at 10,000 r/min for 5 min at 4 °C and collect the supernatant. Concentrate the supernatant under reduced pressure to the aqueous phase. Add 4 mL of redistilled water, then add metaphosphoric acid to a final concentration of 2% (*w*/*v*). Extract three times with 2 mL of ethyl acetate each time. Combine the organic phases, concentrate, and dry at 35–45 °C. Set up three replicates.

First, dissolve the dried sample in 5 mL of 0.1 mol/L HAc. Prior to loading, activate and equilibrate the C18 column with 5 mL of 0.1 mol/L HAc, followed by 5 mL of methanol. Then, load the samples in batches onto a Sep-Pak C18 column. The purification steps are as follows: ① Wash with 5 mL of 17% methanol (methanol: 0.1 mol/L HAc, 17:83, *v*/*v*); ② wash with 5 mL of 40% methanol (methanol: 0.1 mol/L HAc, 40:60, *v*/*v*); ③ elute with 5 mL of 50% methanol (methanol: 0.1 mol/L HAc, 50:50, *v*/*v*) and collect the eluent; ④ elute with 5 mL of 60% methanol (methanol: 0.1 mol/L HAc, 60:40, *v*/*v*) and collect the eluent. Combine the eluents from steps ③ and ④, then concentrate and dry at 40 °C. Dissolve the concentrated, dried sample in methanol and analyze using GC-MS (Thermo Electron Corporation, Boston, MA, USA).

#### 2.3.3. Condensed Tannin Content Determination

The content of condensed tannins was determined using the hydrochloric acid–vanillin method, which was adapted from the method described by Wang et al. (2019) with modifications [29]. All reagents were purchased from Yuanye Reagent Co., Ltd., Shanghai, China.

### 2.4. Data Analysis

Analysis of variance (ANOVA) and Duncan’s multiple range test were performed on the experimental data using SPSS 20.0 and Microsoft Excel 2016. In the Principal Component Analysis (PCA), principal components were selected based on the criteria of the cumulative contribution rate exceeding 70% and eigenvalues greater than 1. Subsequent detailed analysis and plotting were conducted according to the eigenvalues, contribution rates, and cumulative contribution rates of the selected principal components.

## 3. Results

### 3.1. Effects of Insect-Feeding and Leaf-Clipping Stresses on Defense Enzyme Activity in Eucalyptus Leaves

#### 3.1.1. Protein Content in the Tissue of Eucalyptus Leaves Under Different Treatment

**Figure 2:**
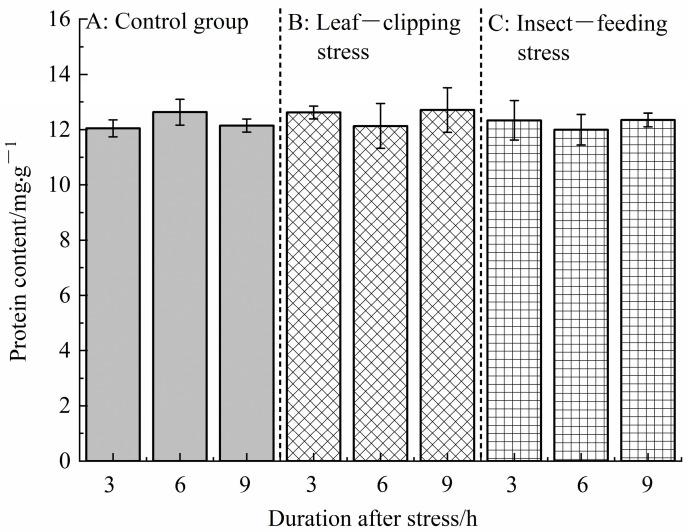
Protein content in the tissue of Eucalyptus leaves under different treatments. (**A**) refers to control group; (**B**) refers to leaf-clipping group; (**C**) refers to insect-feeding group.

To determine the defensive enzyme activities in tissue samples under leaf-clipping stress, pest-feeding stress, and control conditions, the protein content of each sample was first measured. The results showed that there were no significant differences in protein content between the treatment groups and the control group across all tissues (*p* > 0.05) (Figure 2), indicating that the samples could be used for comparative analysis of defensive enzyme activities under different treatment conditions.

#### 3.1.2. Effects of Insect-Feeding and Leaf-Clipping Stresses on Peroxidase Activity in Eucalyptus Leaves

**Figure 3:**
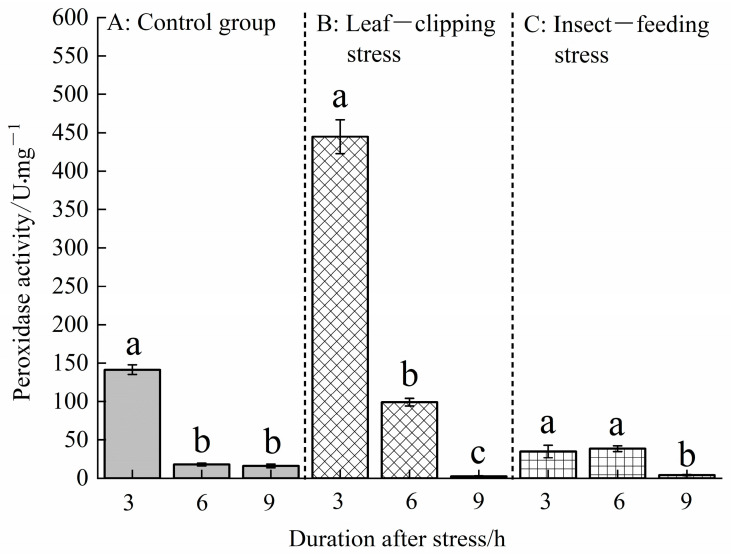
Peroxidase activities at 3, 6, and 9 h in leaves treated with the same stress method. Values are shown as the mean ± SEM. Different letters indicate significant differences in the leaf POD activity between different stress treatments at the α = 0.05 level. (**A**) refers to control group; (**B**) refers to leaf-clipping group; (**C**) refers to insect-feeding group.

Figure 3 shows that there were significant differences in POD activities in *Eucalyptus* leaves under biotic and abiotic stresses (*p* < 0.05). At 3 h post-stress, the POD activity in *Eucalyptus* leaves showed the following order: the leaf-clipping stress had the highest activity at 444.83 U, significantly higher than that in the control; the stimulation caused by the pest-feeding stress was 34.83 U, significantly lower than the control. At 6 h post-stress, the POD activity showed the following order: leaf clipping (99.00 U) > pest feeding (38.50 U) > control (17.83 U). Significant differences were observed between the control, leaf-clipping and pest-feeding stress groups. At 9 h post-stress, the POD activity in the control was significantly higher than those in the pest-feeding and leaf-clipping stress groups.

Under leaf-clipping stress, the POD activity decreased with increasing post-stress duration, showing the following order: 3 h (444.83 U) > 6 h (99.00 U) > 9 h (2.50 U). Under pest-feeding stress, the POD activity initially increased and then decreased over time, showing the following order: 6 h (38.50 U) > 3 h (34.83 U) > 9 h (4.17 U). This indicates that leaf clipping has a more significant inducing effect on peroxidase activity in *Eucalyptus* leaves than pest-feeding stress.

#### 3.1.3. Effects of Insect-Feeding and Leaf-Clipping Stresses on Polyphenol Oxidase Activity in Eucalyptus Leaves

**Figure 4:**
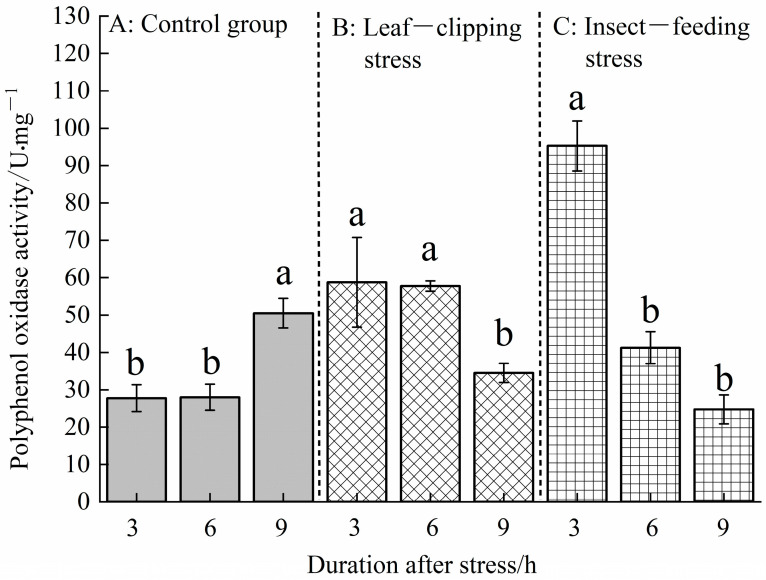
Polyphenol oxidase activities at 3, 6, and 9 h in leaves treated with the same stress method. Values are shown as the mean ± SEM. Different letters indicate significant differences in the PPO activity in *Eucalyptus* leaves between different stress treatments at the α = 0.05 level. (**A**) refers to control group; (**B**) refers to leaf-clipping group; (**C**) refers to insect-feeding group.

Figure 4 shows that there were significant differences in PPO activity in *Eucalyptus* leaves under biotic and abiotic stresses (*p* < 0.05). At 3 h post-stress, the pest-feeding stress had the highest activity at 95.25 U, while the leaf-clipping stress showed an activity of 58.75 U. Both groups differed significantly from the control. At 6 h post-stress, the PPO activities showed the following order: leaf clipping (57.75 U) > pest feeding (41.25 U) > control (28.00 U). Significant differences were observed between the control, leaf-clipping and pest-feeding stress groups. At 9 h post-stress, the polyphenol oxidase activities in the leaf-clipping (34.50 U) and the pest-feeding stress (24.75 U) were lower than that in the control (50.50 U).

Under leaf-clipping stress, the PPO activity decreased with increasing post-stress duration, showing the following order: 3 h (58.75 U) > 6 h (57.75 U) > 9 h (34.50 U). Under pest-feeding stress, the PPO activity also decreased over time, showing the following order: 3 h (95.25 U) > 6 h (41.25 U) > 9 h (24.75 U). In summary, pest-feeding stress has a stronger inducing effect on the polyphenol oxidase activity in *Eucalyptus* leaves than leaf-clipping stress.

#### 3.1.4. Effects of Insect-Feeding and Leaf-Clipping Stresses on Phenylalanine Ammonia-Lyase Activity in Eucalyptus Leaves

**Figure 5:**
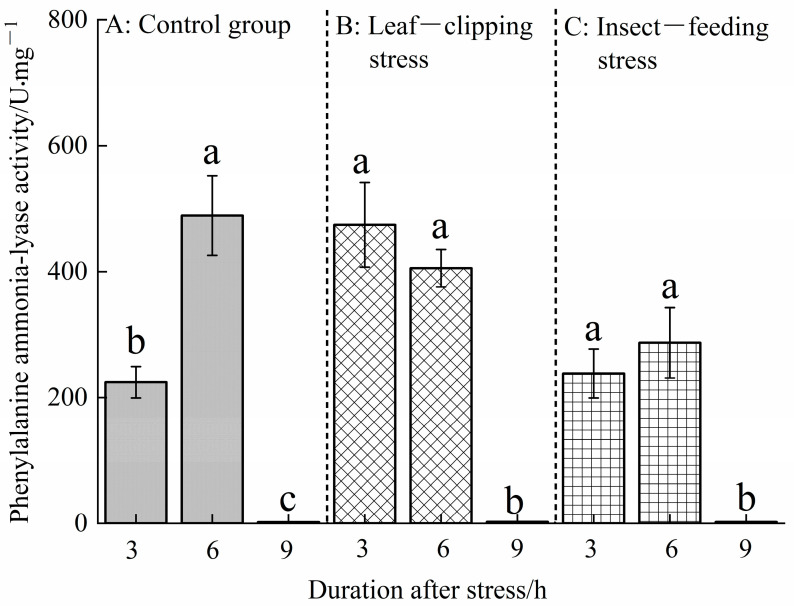
Phenylalanine ammonia-lyase activities at 3, 6, and 9 h in leaves treated with the same stress method. Values are shown as the mean ± SEM. Different letters indicate significant differences in the PAL activity in *Eucalyptus* leaves between different stress treatments at the α = 0.05 level. (**A**) refers to control group; (**B**) refers to leaf-clipping group; (**C**) refers to insect-feeding group.

Figure 5 shows that significant differences were observed in PAL activity in *Eucalyptus* leaves under biotic and abiotic stresses (*p* < 0.05). At 3 h post-stress, PAL activity in Eucalyptus leaves was highest in the leaf-clipping stress (474.38 U), followed by the pest-feeding stress (238.00 U), both of which were higher than that in the control. At 6 h post-stress, PAL activity showed the following order: control (489.00 U) > leaf clipping (405.43 U) > pest feeding (286.86 U); a significant difference was observed between the control and pest-feeding stress groups.

Under leaf-clipping stress, the PAL activity in *Eucalyptus* leaves gradually decreased over time, with the order being 3 h (474.38 U) > 6 h (405.43 U) > 9 h (2.33 U) after stress. Under pest-feeding stress, the order was 6 h (286.86 U) > 3 h (238.00 U) > 9 h (2.13 U) after the stress. In summary, these results indicate that leaf clipping has a stronger inducing effect on PAL activity in *Eucalyptus* leaves than pest-feeding stress.

### 3.2. Effects of Insect-Feeding and Leaf-Clipping Stresses on the Contents of Defense Compounds in Eucalyptus Leaves

#### 3.2.1. Temporal Changes in Salicylic Acid Content Induced by Insect-Feeding and Leaf-Clipping Stresses

**Figure 6:**
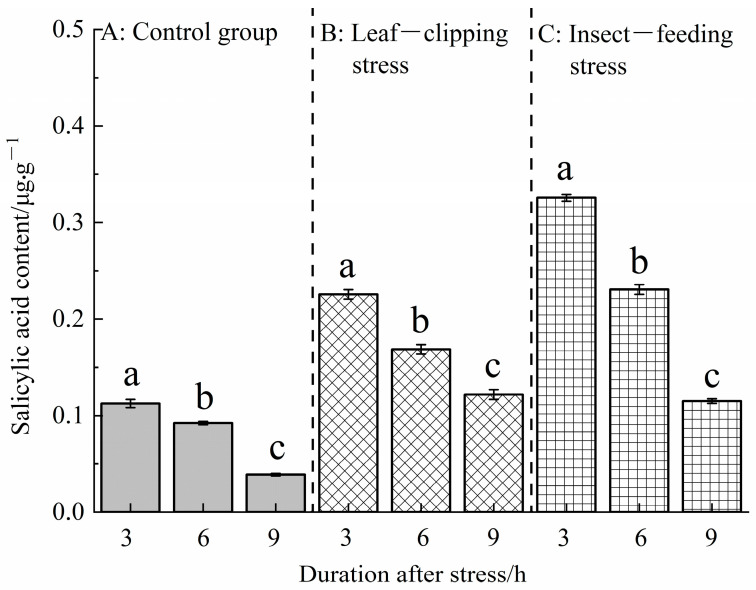
Salicylic acid contents at 3, 6, and 9 h in leaves treated with the same stress method. Values are shown as the mean ± SEM. Different letters indicate significant differences in the salicylic acid contents in leaves between different stress treatments at the α = 0.05 level. (**A**) refers to control group; (**B**) refers to leaf-clipping group; (**C**) refers to insect-feeding group.

Figure 6 shows that there were significant differences in SA content of *Eucalyptus* leaves under different stress treatments at the same time point after stress (*p* < 0.05). At 3 and 6 h after stress, the SA contents showed the following order: pest-feeding stress > leaf-clipping stress > control. At 9 h after stress, the SA content showed the following order: leaf-clipping stress > pest-feeding stress > control; there was no significant difference between the leaf-clipping and pest-feeding stress groups.

SA contents also showed significant differences at different time points (3, 6, and 9 h) after the same stress treatment (*p* < 0.05). The control, leaf-clipping and pest-feeding stress groups all exhibited the same trend: SA content at 3 h after stress > 6 h > 9 h; there was a significant difference between each pair of time points. The results show that leaf-clipping and pest-feeding stress can induce the synthesis and accumulation of salicylic acid in *Eucalyptus* leaves.

#### 3.2.2. Temporal Changes in Jasmonic Acid Content Induced by Insect-Feeding and Leaf-Clipping Stresses

**Figure 7:**
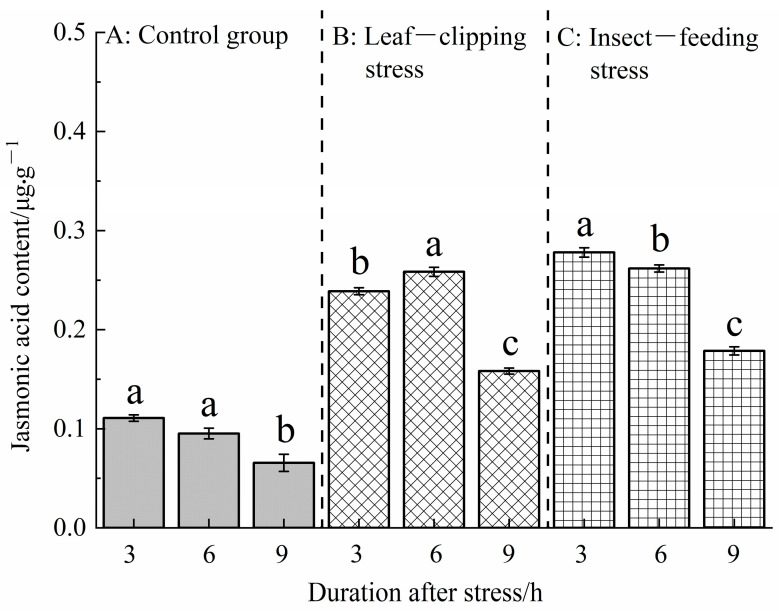
Jasmonic acid contents at 3, 6, and 9 h in leaves treated with the same stress method. Values are shown as the mean ± SEM. Different letters indicate significant differences in jasmonic acid contents in leaves between different stress treatments at the α = 0.05 level. (**A**) refers to control group; (**B**) refers to leaf-clipping group; (**C**) refers to insect-feeding group.

Figure 7 shows that there were significant differences in JA contents of *Eucalyptus* leaves under different stresses at the same time point after stress (*p* < 0.05). At 3 and 9 h after stress, JA contents showed the following order: pest-feeding stress > leaf-clipping stress > control; there were significant differences between each group. At 6 h after stress, there was no significant difference between the leaf-clipping and pest-feeding stress groups, but both were significantly higher than the control.

JA contents also showed significant differences at different time points (3, 6, and 9 h) after the same stress treatment (*p* < 0.05). The control and pest-feeding stress groups exhibited the same trend: JA content at 3 h after stress > 6 h > 9 h. The leaf-clipping stress showed a different trend: JA content at 6 h after stress > 3 h > 9 h. Similarly, leaf-clipping and pest-feeding stress significantly promoted the synthesis of jasmonic acid in *Eucalyptus* leaves.

#### 3.2.3. Temporal Changes in Tannin Content Induced by Insect-Feeding and Leaf-Clipping Stresses

**Figure 8:**
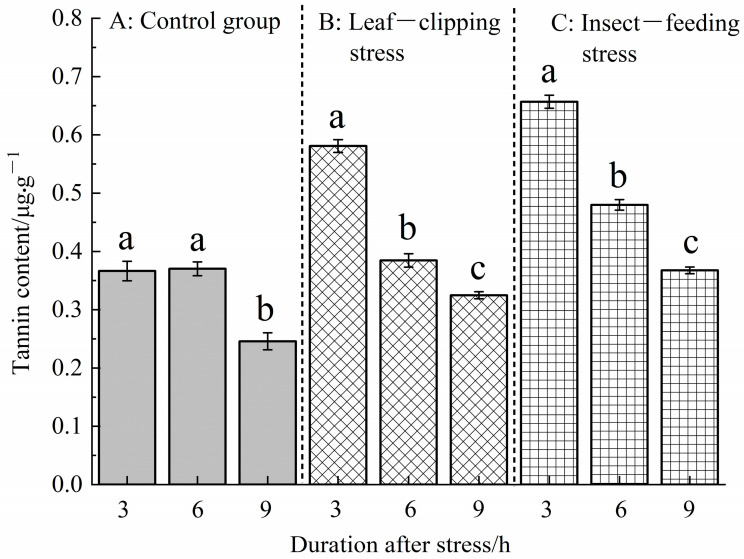
Tannin contents at 3, 6, and 9 h in leaves treated with the same stress method. Values are shown as the mean ± SEM. Different letters indicate significant differences in the leaf tannin content between different stress treatments at the α = 0.05 level. (**A**) refers to control group; (**B**) refers to leaf-clipping group; (**C**) refers to insect-feeding group.

Figure 8 shows that there were significant differences in the tannin content of *Eucalyptus* leaves under different stresses at the same time point after stress (*p* < 0.05). At 3, 6, and 9 h after stress, the tannin contents consistently showed the following order: pest-feeding stress > leaf-clipping stress > control. At 6 h, there was no significant difference between the control and leaf-clipping stress groups, but all other pairwise comparisons showed significant differences.

The tannin contents also showed significant differences at different time points (3, 6, and 9 h) after the same stress treatment (*p* < 0.05). The control group exhibited the following trend: tannin content at 6 h after stress > 3 h > 9 h; there was no significant difference between 6 and 3 h, but both were significantly higher than at 9 h. The trend in the leaf-clipping and pest-feeding stress groups was as follows: tannin content within 3 h after stress > 6 h > 9 h. From the above results, the leaf-clipping and pest-feeding stress have significant effects on tannin synthesis in *Eucalyptus* leaves within a short period after stress.

### 3.3. Principal Component Analysis of the Defense Responses of Eucalyptus Leaves

**Figure 9:**
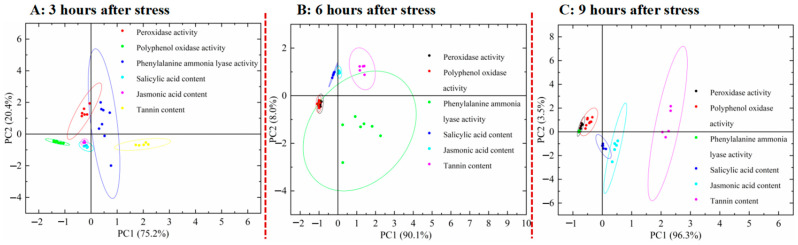
Scree plots of the ecological adaptability of *E. urophylla* × *E. grandis* under different stress conditions. (**A**) refers to 3 h after stress; (**B**) refers to 6 h after stress; (**C**) refers to 9 h after stress.

Figure 9 shows that POD activity and salicylic acid content are the primary factors in the defensive responses of *Eucalyptus* to pest-feeding and leaf-clipping stresses. At 3, 6, and 9 h after exposure to biotic and abiotic stresses, POD activity had the highest eigenvalue, followed by the salicylic acid content, while the tannin content exhibited the lowest eigenvalue. At three different durations after exposure to stress, POD activity (first principal component) demonstrated the highest contribution rate, while the salicylic acid content (second principal component) also showed a relatively high contribution. The cumulative contribution rate of these two principal components (POD activity and salicylic acid content) exceeded 95%, whereas the contribution rate of tannin content was the lowest. This indicates that POD and salicylic acid play important roles in *Eucalyptus* responses to biotic and abiotic stresses.

## 4. Discussion

*Eucalyptus* is primarily distributed across various provinces in southern China and serves as a highly significant timber species in the country. An outbreak of the pest *B. suppressaria*, which extensively feeds on *Eucalyptus* leaves, often results in complete defoliation. This severely compromises photosynthesis in *Eucalyptus* and leads to substantial economic losses [14]. This study systematically investigated the defensive responses of *Eucalyptus* to biotic and abiotic stresses, aiming to provide a theoretical foundation for insect-resistant breeding and integrated pest management strategies in *Eucalyptus*.

In the long-term co-evolution of plants and their environment, plants activate a series of sophisticated stress responses when exposed to external biotic or abiotic stimuli. The core mechanism of this induced resistance lies in regulating internal physiological and biochemical processes, such as the activity of key enzyme systems and the synthesis of secondary metabolites, thereby systematically enhancing the plant’s stress tolerance [30]. Different inducing factors can activate distinct defense signaling pathways, leading to specific physiological effects. In this process, key defense enzymes, such as peroxidase (POD), serve as critical regulatory hubs, playing an indispensable role in plant defense responses to various stresses [31]. In this experiment, the POD activities in the control group at 3 and 6 h were lower than those in the leaf-clipping and pest-feeding stress groups, indicating that POD activity functions in the short term to assist plants in resisting external stresses. Previous studies have shown that mechanical damage is a purely physical stimulus, with a simple yet intense response process, whereas insect feeding involves more complex mechanisms. Due to the influence of insect saliva, plants experience not only physical but also chemical stimulation [32]. In this experiment, the POD activities in the leaf-clipping group at 3 and 6 h were higher than that in the pest-feeding stress, suggesting that plants respond more rapidly and intensely to clipping stress in the short term. In contrast, stimulation by pest secretions may trigger a more complex defense response in *Eucalyptus* leaves. The leaf-clipping and pest-feeding stress treatments exhibited an initial increase followed by a decline in POD activity over time. Previous research has found that plant resistance is relative; under appropriate induction conditions, plants develop induced resistance. However, once a certain threshold is exceeded, plant resistance may decrease with increasing stress intensity [33].

Plants do not continuously or excessively express defense compounds. Instead, they selectively, intermittently, and efficiently adjust their expression in response to changes in external stimuli. The concentration of POD often increases rapidly in the short term but gradually returns to normal levels—or may even fall below baseline—over time [34]. In this experiment, the POD activities in the leaf-clipping and pest-feeding stress groups initially increased and then declined, with the activity at 9 h lower than that in the control group. A comparison between the leaf-clipping and pest-feeding groups revealed that the rate of change in POD activity was significantly higher in the leaf-clipping group. This discrepancy may be attributed to the fact that leaf clipping induces a rapid, mechanical simulation of insect damage, whereas insect feeding triggers a more complex suite of plant defense responses. Studies on the role of insect salivary components in plant–insect interactions have suggested that clipping primarily involves mechanical damage as a physical stimulus, whereas feeding combines physical damage from insect mandibles with chemical stimulation from oral secretions, resulting in a more intricate and prolonged effect [35,36].

At 3 and 6 h after stress, the polyphenol oxidase (PPO) activities in the pest-feeding and leaf-clipping stress groups were higher than in the control group. This indicates that chemical and mechanical stimuli triggered by pest feeding and leaf clipping activate defense responses in plant cells. At 9 h, the PPO activity in the control group was higher than in the stress groups, likely due to the gradual enhancement of the plant’s defense mechanisms and increased resistance to external stress over time. Previous studies have found that after feeding by *Clostera anachoreta* larvae, the PAL, LOX, and PPO activities in damaged leaves and adjacent leaves of *Populus canadensis* were significantly higher than in the control group. This indicates that plants not only exhibit localized defense responses at the site of damage but also develop systemic resistance in undamaged tissues or even the entire plant. In leaves adjacent to those fed on by pests, the PPO activity also increased significantly and rose progressively over time, suggesting the induction of systemic resistance in *P. canadensis* [37]. In this experiment, the leaf-clipping and pest-feeding stress treatments exhibited a gradual decline in PPO activity over time. The plant’s response to insect feeding demonstrates an “on–off effect,” where the defense system activates when external stimuli reach a certain threshold, and deactivates or diminishes when the stimuli exceed a specific range [38]. This phenomenon aligns with the “optimal defense theory” or “defense cost hypothesis,” where the sustained or excessive activation of defenses can lead to resource depletion or feedback inhibition of signaling pathways, resulting in a decline in specific enzyme activities [39,40].

Compared with the control group, the phenylalanine ammonia-lyase (PAL) activity in *Eucalyptus* leaves subjected to pest-feeding and leaf-clipping stresses significantly increased, indicating that this enzyme plays a positive role in the plant’s response to mechanical damage. This finding is consistent with responses observed in other plants under similar stress conditions. For instance, leaf-clipping treatment has been shown to induce a rapid increase in PAL activity in *Larix gmelinii*, maintaining it at a high level that was significantly different from the control group [41].

The synthesis of plant secondary metabolites depends on the consumption of the plant’s own nutrients. To maintain normal physiological functions, growth, and the homeostasis of defense systems, the secondary metabolite levels often rise temporarily before gradually returning to baseline. This explains, in part, the decline in defense enzyme activities, such as PPO, over time in this experiment [39]. In the leaf-clipping treatment, salicylic acid (SA) content in *Eucalyptus* leaves peaked at 3 h and did not continue to decline over time. However, under different clipping intensities (25%, 50%, and 70% of four branches), the SA content generally showed a decreasing trend from 0.5 to 120 h, with similar patterns observed for treatments involving eight and twelve branches. As a single form of physical damage, mechanical stress elicits a relatively simple and stable response, inducing significant changes in plant leaves in the short term. However, as the stimulus diminishes and the plant’s defense system self-regulates, SA levels eventually stabilize.

Compared with the control and leaf-clipping groups, the pest-feeding stress induced smaller fluctuations in the PAL activity. Plant resistance is a complex process coordinated by multiple defense enzymes and secondary metabolites, and different types of stress may activate distinct defense pathways [41]. In this experiment, the SA content in the pest-feeding stress was lower than in the control group at 3 and 6 h. This may indicate that other defense signaling pathways, such as the jasmonic acid pathway, play a more dominant role in the early response of *Eucalyptus* to insect feeding [42], while PAL is not a core enzyme in its anti-insect response. Consequently, PAL activity fluctuates relatively mildly under biotic stress. This observation aligns with findings from some studies, suggesting that plant defense responses to different stress sources are specific and pathway-dependent [40]. SA treatment can rapidly increase the PAL activity in peanut leaves to a peak before it declines [43]. In this experiment, the PAL activity in *Eucalyptus* leaves showed a similar trend over time, eventually stabilizing. This follows the general pattern of dynamic changes in enzyme activity during induced plant defense: rapid activation of the defense response in the early stages of stress, followed by gradual restoration of homeostasis through feedback regulation to avoid the metabolic burden of sustained activation [39].

In addition to causing physical damage, biotic stress involves chemical induction, making the process more complex than leaf clipping. It involves dynamic defense and counter-defense mechanisms between plants and insects [41]. Several studies have noted that PAL activity may exhibit multi-peak fluctuations after plants are subjected to biotic stress [38]. In this experiment, the detected PAL activity in *Eucalyptus* leaves at 9 h was 2.13 U. It is speculated that this time point might have coincided with a critical period of peak activity or a rapid increase phase. Due to the rapid enzyme reaction or limitations in the detection environment and equipment, the enzyme activity signal may not have been effectively captured. As a key enzyme in the phenylpropanoid pathway, PAL plays an important role in the immediate and delayed induced defenses in *Larix gmelinii* [41]. In this experiment, the PAL activity in the pest-feeding stress remained lower than in the control and leaf-clipping groups at 3 and 9 h, possibly indicating a delayed induced defense in *Eucalyptus* after insect damage [40]. Notably, the PAL activity in *Eucalyptus* leaves in the control group at 6 h was significantly higher than in the leaf-clipping and pest-feeding stress groups. This may stem from the immediate activation and dynamic adjustment of the plant’s defense mechanisms, reflecting the temporal and complex nature of induced defense [42].

Jasmonic acid (JA) is a key signaling molecule in plant defense responses. It can directly deter insect feeding through increased levels and systemically induce defense responses in undamaged tissues [44]. It is currently believed that chemical elicitors in the oral or oviposition secretions of herbivorous insects can activate signaling pathways in plants during feeding or oviposition [45]. For example, oral secretions from *Spodoptera frugiperda* can induce increased ethylene, JA, and SA levels in host plants and promote the release of volatile compounds to attract natural enemies [46]. Feeding by *Tuta absoluta* larvae can also enhance SA and JA signaling in host plants, thereby increasing the defense enzyme activity, secondary metabolite content, and protease inhibitor activity [47]. The plant-induced defense can affect insect feeding on hosts. Based on this study, it was found that there is a specific coevolution between plants and insects, which provides resistance improvement strategies for plants [48]. These mechanisms provide important insights for utilizing plant-induced resistance to manage defoliating pests.

Enzymes and plant secondary metabolite defensive compounds play important roles in plant responses to external stresses. For example, phenolic compounds and tannins are frequently accumulated in plant leaves following insect feeding, which can reduce palatability and interfere with insect digestion [49]. Increased activities of peroxidase (POD) and polyphenol oxidase (PPO) can lead to the oxidation of phenolic compounds and the cross-linking of cell wall proteins, thereby enhancing physical barriers and reducing nutrient availability to herbivores [50]. In this study, enzymes and secondary metabolite defensive compounds exhibited significant effects in Eucalyptus during responses to both biotic and abiotic stresses, indicating that enzyme- and secondary metabolite-mediated defense responses are widespread and highly conserved across plant species.

However, the current study was designed as a focused physiological investigation to establish a foundational understanding of the immediate defense responses (enzyme activities and secondary metabolites) against specific stresses. Future studies will employ multi-omics approaches (e.g., transcriptomics and proteomics) to elucidate the regulatory networks linking gene expression to these physiological changes. While this study focuses on a single model interaction, future research will expand to include comparative analyses across different Eucalyptus species or ecotypes with varying susceptibilities, as well as other herbivorous insects, to validate the generality of our findings and explore resistance mechanisms more broadly.

## 5. Conclusions

Insect-feeding and leaf-clipping stresses significantly affect the peroxidase (POD), polyphenol oxidase (PPO), and phenylalanine ammonia-lyase (PAL) activities and also affects the content of jasmonic acid (JA), salicylic acid (SA), and tannin in Eucalyptus leaves. The POD activity and salicylic acid content were identified as the primary factors in *Eucalyptus* responses to pest-feeding and leaf-clipping stresses. Therefore, *Eucalyptus* exhibits significant defense responses to biotic and abiotic stresses, demonstrating its ability to adapt effectively to such environmental challenges.

## Data Availability

The original contributions presented in this study are included in the article. Further inquiries can be directed to the corresponding authors.
